# Remediation of the picture‐text problem for learners exhibiting reading deficits

**DOI:** 10.1002/jaba.70001

**Published:** 2025-02-10

**Authors:** Taylor K. Lewis, Tom Cariveau

**Affiliations:** ^1^ Department of Psychology University of North Carolina Wilmington Wilmington NC USA

**Keywords:** compound stimuli, overselectivity, picture‐book reading, picture‐text problem, stimulus control

## Abstract

Early reading materials are replete with pictures. Pictures purportedly improve reading comprehension and motivation; however, the simultaneous presentation of pictures and text can also impede textual control for some readers. Attempts to remediate restricted stimulus control in picture–text compounds suggest that omitting the picture element is most effective, although these arrangements may also be less socially valid. The current study is an evaluation of a novel compound stimulus prompt (CSP) arrangement that required that the learner differentially respond to the underselected (i.e., textual) element during picture‐book reading. The development of textual control in this condition was compared with that in text‐only and picture prompt arrangements. The CSP condition required the same or fewer sessions to produce textual control as the text‐only condition for five out of six participants who exhibited reading difficulties. Participants emitted more correct responses during CSP and picture prompt instruction and preferred these conditions to the text‐only condition during a concurrent‐chains assessment.

From early childhood, children are exposed to reading materials that are replete with pictures or illustrations. Pictures purportedly contribute to greater reading comprehension (Brookshire et al., 2002; Mastropieri & Scruggs, 1996; Rusted & Coltheart, 1979) and motivation to read (Carney & Levin, 2002; Concannon, 1975; McGeown et al., 2020; Samuels et al., 1974); however, pictures have also been shown to hinder reading performance under certain conditions (Samuels, 1967, 1970). Specifically, the simultaneous presentation of pictures and text has been shown to impede control by the textual stimulus (Didden et al., 2000; Dittlinger & Lerman, 2011; Richardson et al., 2017; Samuels, 1967; Singh & Solman, 1990; see review by Kennedy & Cariveau, 2023). As a result, early reading experiences may commonly result in a learner's responding coming under the control of the illustrations rather than the text, termed the picture‐text problem.

The picture‐text problem is perhaps best conceptualized as an instance of restricted stimulus control (Dittlinger & Lerman, 2011; Singh & Solman, 1990). Singh and Solman (1990) were the first to provide such a conceptualization when describing the impeding effects of picture–text compounds on the development of textual control for eight children with intellectual disabilities. The authors described their findings as an example of the picture *blocking* control by the textual stimulus because their participants could name the picture but not the corresponding textual element of the compound stimulus prior to instruction. Indeed, *stimulus blocking* occurs when a differential history with an individual element of the compound stimulus impedes control by the other element(s) (Johnson & Cumming, 1968; Seraganian & vom Saal, 1969; Singh & Solman, 1990). It is also possible that the picture‐text problem is an example of *stimulus overshadowing*, characterized by the development of restricted stimulus control despite no differential history with any element of the compound (e.g., the picture overshadows control by the text). For example, Dittlinger and Lerman (2011) arranged picture–text compounds that included known or unknown pictures and observed diminished textual control in this condition relative to that in a text‐only condition regardless of the participants' tact performance before instruction.

Given the preponderance of examples of the picture‐text problem (Kennedy & Cariveau, 2023), instructors may simply remove illustrations from materials intended to produce responding under textual control. However, given the ubiquity of pictures in early reading materials (Hodkinson, 2017), text‐only instruction may be untenable, or at least unpopular, among learners and educators. As a result, several studies have investigated methods to mitigate the picture‐text problem while retaining the picture element (e.g., Richardson et al., 2017; Saunders & Solman, 1984; Singh & Solman, 1990; Solman et al., 1992). Some strategies described in the extant literature include reducing the size of the picture relative to the text (Didden et al., 2000; Singh & Solman, 1990; Solman et al., 1992), embedding the textual stimulus within the picture and adjusting the brightness of the picture element (Richardson et al., 2017), or presenting the textual stimulus before the picture (Saunders & Solman, 1984). These attempts have generally failed in remediating the picture‐text problem such that text‐only arrangements commonly resulted in greater, or more rapid development of, textual control (Didden et al., 2000; Richardson et al., 2017; Singh & Solman, 1990; Wu & Solman, 1993). Importantly, certain procedures also require the development of several supplemental materials (e.g., pictures of varying brightness in text‐picture fading; Richardson et al., 2017) or instruction targeting other relations, such as arbitrary conditional discriminations (e.g., picture‐text matching; Richardson et al., 2017; Wu & Solman, 1993), which may be challenging for some learners (Eikeseth & Smith, 1992; Pilgrim et al., 2000; Richardson et al., 2017). As a result, additional research is needed to identify effective methods to mitigate the picture‐text problem.

In the picture‐text problem, the textual element may be considered the underselected component of the compound stimulus (Broomfield et al., 2008). Given that the picture‐text problem is characterized by a lack of control by all relevant features of a compound, requiring that the learner differentially respond to the underselected (i.e., textual) element may produce greater control by that element. This may be accomplished by arranging a differential observing response, which requires discrimination of the relevant stimulus features before a learner emits the target response (Farber & Dickson, 2023). Previous research has found that including differential observing responses during matching‐to‐sample tasks with compound sample stimuli produced greater control by the underselected element (Dube & McIlvane, 1999; Farber et al., 2017; Walpole et al., 2007). To date, no previous research seeking to remediate the picture‐text problem has required that the learner differentially respond to the textual element of the compound.

The current study extended previous research on the picture‐text problem by evaluating the effect of three experimental conditions on textual responding during book reading activities. The three conditions were text‐only, picture prompt, and compound stimulus prompt (CSP) arrangements. The latter condition included an array of picture–text compounds that required the participants to differentially respond to the textual element of the compound stimulus. The current study extended previous research on the picture‐text problem by (a) evaluating the effects of the experimental conditions during picture‐book reading rather than during instruction with flashcards and (b) measuring participants' preference for the experimental conditions using a concurrent‐chains assessment.

## METHOD

### Participants, setting, and materials

Six students attending a high‐poverty elementary school (McFarland et al., 2019) participated. Each participant was identified by their teacher as having the highest need among their classmates for a reading intervention. Each participant's legal guardian provided permission to participate, and participants provided vocal assent daily. All participants identified as Black. Avina, Holly, Beau, and Jalani were all enrolled in first grade. Holly and Avina were girls. Beau and Jalani were boys. Salem was a girl enrolled in second grade. Ivy was a girl in third grade. None of the participants received special education services. The experimenter assessed each participant's word reading performance using the Dynamic Indicators of Basic Early Literacy Skills Word Reading Fluency benchmark assessment (University of Oregon, 2020). All participants' performance fell in the lowest performing (i.e., intensive support) category on the assessment.

All sessions were conducted in a 1:1 arrangement in a classroom at the participants' elementary school. Each participant sat with an experimenter at a table at least 2 m from other participants to minimize distractions.

The experimenter used data collection materials during sessions including individualized data sheets, pens, timers, and clipboards. We printed separate instructional booklets for each condition on half sheets of white paper (21.59 × 13.97 cm). Each booklet included a title page, eight instructional pages, and a probe page. The title page and all instructional pages included a background picture (e.g., an image of a park or house) and a cartoon‐style image of a character (e.g., a person or animal). Background pictures were included to mimic common features of children's books. Each page in a booklet included the same background picture and character, but the background and character differed across conditions. When representational pictures of target textual stimuli were included (i.e., picture prompt and CSP conditions), they appeared in a small white box over the background picture. All background pictures, characters, and representational pictures were prepared using Canva, an online design platform (www.canva.com). During instruction, sentences with target and nontarget textual stimuli appeared below the background picture on a white background in black, size‐30 Comic Sans font. During teaching and mastery probes, target and nontarget textual stimuli were presented on a probe page. This page included two columns of four words in black, size‐50 Comic Sans font on a white background. All concurrent‐chains preference assessment sessions were conducted on a tablet using the Microsoft PowerPoint application.

### Dependent variable

Trained data collectors recorded correct and incorrect responses. A correct response was recorded if the participant emitted a predefined response that corresponded to the presented textual stimulus within 5 s of the stimulus being presented. An incorrect response (i.e., error) was scored if the participant emitted any response other than the correct target response or did not emit any response within 5 s.

Time to mastery was calculated separately across conditions. Trained data collectors recorded the duration of each instructional session. The data collector started a count‐up timer once the participant read the first word of the title and stopped the timer once the participant read the final word on the last instructional page. Total time to mastery was calculated by summing the duration of all instructional sessions.

### Design

We used an adapted alternating‐treatments design to compare the effects of each teaching condition on textual responding (Sindelar et al., 1985). Based on the recommendations of Cariveau et al. (2021), four equated targets were assigned to each condition. Specifically, target sets were equated based on the number of letters and syllables. Within each target set, each target started with the same letter as one other target in the set. That is, two targets shared the same first letter and the remaining two targets shared a different first letter. For example, a set may include targets “leaf,” “lion,” “baby,” and “bear.” Targets beginning with the same first letter were assigned to the same condition to reduce the potential development of control by only the first letter of the target textual stimuli. No words across conditions began with the same first letter. Each evaluation included a target set assigned to a no‐instruction control condition to detect possible threats to internal validity (e.g., historical threats; Cariveau & Fetzner, 2022).

### Preassessment

During preassessment, the experimenter presented potential textual targets individually and recorded the participant's response. Textual stimuli that the participant did not correctly read during the textual preassessment were included in the tact preassessment. During the tact preassessment, the experimenter presented a picture of each potential target and instructed the participant to tact the stimulus. We assigned a target to one of the experimental conditions only if the participant responded incorrectly to the textual stimulus and correctly to the corresponding picture stimulus. Nontarget stimuli to be used in the book titles and sentence frames were selected based on word lengths that were appropriate for the participant's reading level. The titles and sentence frames included mastered and unmastered textual stimuli.

### Baseline

The experimenter presented the probe page for each condition and instructed the participant to read each word. Eight words appeared on the probe page. Four nontarget textual stimuli that appeared in the title or sentence frame of the instructional booklet were interspersed among the four target textual stimuli. The order of textual stimuli on the probe page varied across sessions. Responses to the target textual stimuli during baseline probes produced no differential consequences (e.g., “ok” or “keep going”). Correct responses to the nontarget textual stimuli produced praise, and incorrect responses to the nontarget textual stimuli produced echoic prompts.

### General procedure

Separate booklets were prepared for each condition such that one booklet included targets from a single experimental condition. The experimenter presented each instructional booklet (i.e., condition) a single time each day, and the order of conditions varied across days. Each booklet included four target textual stimuli, each of which appeared as the target on two separate pages for a total of eight instructional pages per booklet. Each target appeared once in the first and second halves of the booklet. The pages of the booklet were shuffled before each instructional session. Each page of the teaching booklet included a sentence frame followed by the target textual stimulus (e.g., “The boy felt the [target]”). The sentence frame was the same for all pages in a condition but differed across conditions. Each sentence frame included the same general structure (i.e., “The [noun] [verb] the [target]”).

Trained graduate students implemented all experimental procedures under the supervision of a doctoral‐level Board Certified Behavior Analyst. Instructional sessions began with the experimenter placing the booklet in front of the participant and instructing them to read the book. Incorrect responses to any textual stimulus in the sentence frame (i.e., nontarget textual stimuli) resulted in an echoic prompt. Correct responses to the target textual stimulus resulted in praise, and incorrect responses resulted in condition‐specific consequences (described below).

Once the participant read all eight instructional pages, the experimenter immediately presented a text‐only teaching probe. All probe procedures were identical to baseline, and the order of targets on the probe page differed each day. If the participant emitted 100% unprompted correct responses to the target textual stimuli during a teaching probe, a mastery probe was conducted the following school day before instruction for any condition. Mastery probe procedures were identical to teaching probes and only differed in that they occurred before instruction for that day. Instruction continued until the participant emitted 100% unprompted correct responses on consecutive teaching and mastery probes.

### Compound stimulus prompt condition

**FIGURE 1:**
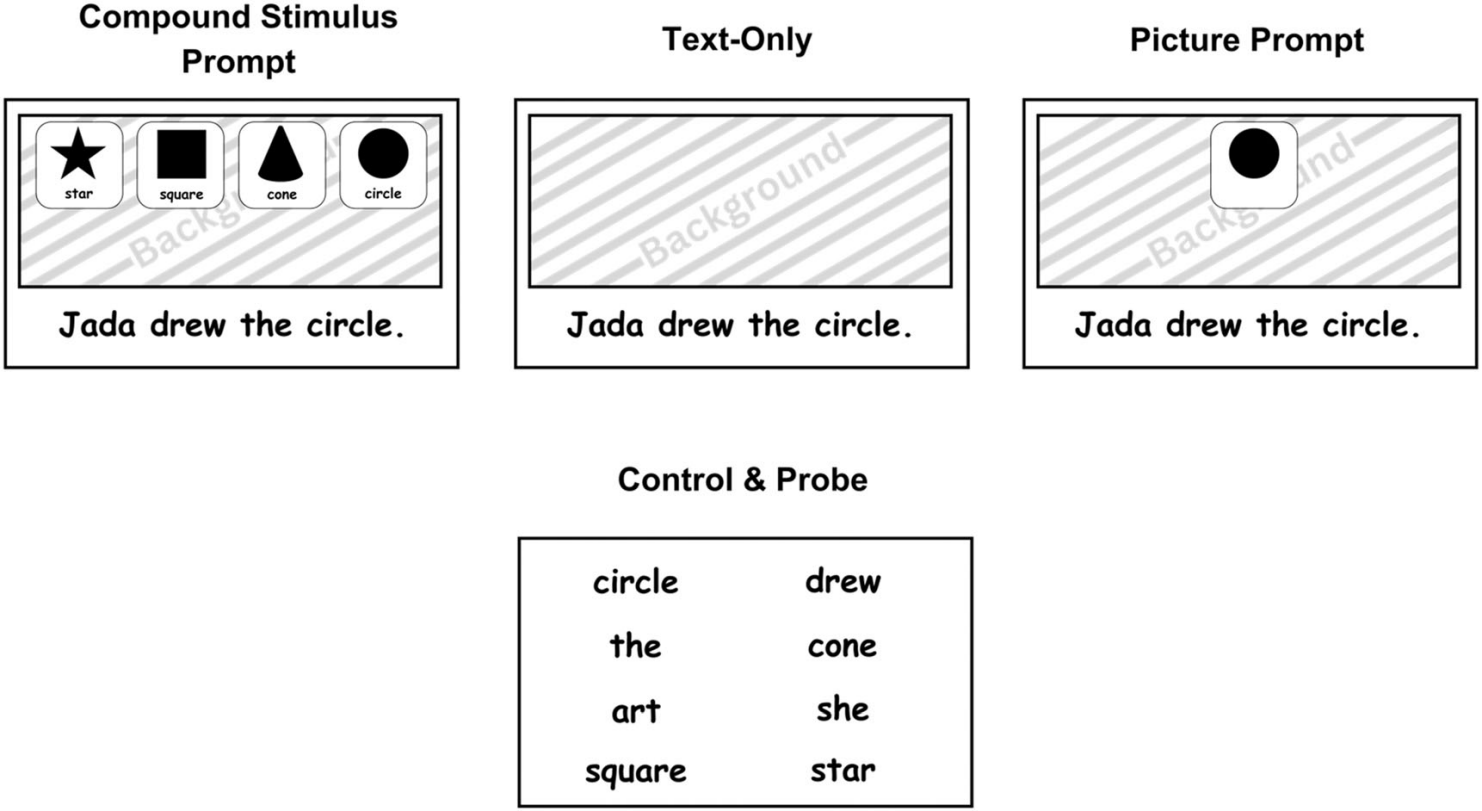
Example pages from each comparison condition and probes. Unique targets were assigned to conditions. A single target is shown as an example of each condition. A probe page appeared as the final page of each book.

Figure 1 shows an example page from the CSP condition. Each page in this condition included an array of picture–text compound stimuli at the top of the page. The textual stimulus in each picture–text compound appeared directly below a representative picture. The compound stimulus array consisted of four picture–text compounds, one for each target in the set. The picture–text compound array required that the participant differentially respond to the textual element of the compound to respond correctly. Specifically, although no overt matching response was required before the participant emitted the correct textual response, we hypothesized that the CSP arrangement required the participant to match the textual stimulus in the sentence to the identical textual element in the CSP array (e.g., textual stimulus “circle”), which would then allow the corresponding picture (e.g., picture of a circle) to occasion the correct target response (e.g., vocal response “circle”). According to this conceptualization, the picture can be said to function as a tact prompt. If the participant emitted an incorrect response, the experimenter instructed them to look at the pictures, but did not gesture to any specific picture–text compound, and the data collectors recorded an incorrect response. If the participant emitted another error, an echoic prompt was delivered and the participant was required to echo the prompt before progressing to the next page. The order of the compound stimuli in the comparison array was counterbalanced across trials so that each picture–text compound appeared in each position of the array an equal number of times. Additionally, the position of the S+ compound stimulus appeared in each position in the array an equal number of times within a booklet and the same picture–text compound appeared as the S+ in different positions of the array.

### Text‐only condition

Figure 1 shows an example text‐only instructional page, which included targets presented at the end of the sentence frame with no corresponding picture on the page. Correct responses to the target textual stimulus produced praise. Incorrect responses to the target textual stimulus produced an echoic prompt. The participant was required to echo the prompt before progressing to the next page.

### Picture prompt condition

Figure 1 shows an example page from the picture prompt condition, in which all pages included a single picture that corresponded to the target textual stimulus on each page. Correct responses produced praise and the presentation of the next page. If the participant responded incorrectly, the experimenter instructed the participant to look at the picture and the data collector recorded an incorrect response. If the participant emitted another incorrect response, the experimenter delivered an echoic prompt. The participant was required to echo the prompt before progressing to the next page. This condition was included to assess whether the simultaneous presentation of pictures and text impaired textual responding during text‐only (i.e., teaching and mastery) probes consistent with the picture‐text problem.

### Control

We assessed the participant's responding to a set of four targets that were not exposed to instruction using baseline procedures. The control targets appeared on a probe page like those prepared for the experimental conditions.

### Remedial instruction (Beau and Jalani only)

Consistent with the recommendations of Kodak and Halbur (2021), a discontinuation criterion was set to prevent prolonged exposure to ineffective instructional conditions. Following mastery of the two most efficient conditions, participants were exposed to no more than 150% of instructional sessions required to produce responding at the mastery criterion in the second most efficient condition. If a participant met the discontinuation criterion for a condition, the targets from that condition were presented using procedures from the condition that produced mastery in the fewest number of sessions for that participant.

### Concurrent‐chains preference assessment

We assessed each participant's preference for the instructional conditions after their responding met the mastery criterion for all three conditions. Preference was assessed using a concurrent‐chains arrangement. Five initial links were presented to each participant. We prepared five novel booklets, which were presented on a tablet using a Microsoft PowerPoint slideshow. During each initial link, the experimenter presented the participant with a slide showing three images that represented a single page from the picture prompt, CSP, and text‐only conditions of a new booklet. The experimenter told the participant that they would read a new book and that the participant could select the book type. The experimenter then gestured to each condition‐specific page, briefly described the corresponding condition, and instructed the participant to choose one of the pages. The participant's selection of a condition‐specific page served as the initial link. The participant then read the version of the new booklet using the condition‐specific procedures. This procedure was repeated with each of the five novel booklets for each participant. One or two initial links were presented each day. If two initial links were presented in a single day, they were separated by at least one other reading activity.

### Interobserver agreement and procedural fidelity

Two independent observers were present during at least 42.86% of teaching sessions per participant (*M* = 66.36%) and 39.13% of probe sessions per participant (*M* = 52.17%). Trial‐by‐trial interobserver agreement was calculated following each session. Each presentation of a target textual stimulus was recorded as a trial. An agreement was scored if both observers recorded the same response for a trial. Agreement was calculated by dividing the number of trials with an agreement by the total number of trials and multiplying by 100. Mean agreement was 98.91% (range: 87.50%–100%) during teaching sessions and 99.26% (range: 75%–100%) during probe sessions. We also calculated interobserver agreement for duration of instructional sessions. An agreement was scored if the observer's reported session durations differed by fewer than 2 s. Duration agreement was 100%. A second observer was also present during at least 20% (*M* = 60%) of initial links during the concurrent‐chains preference assessment for each participant. An agreement was recorded if both observers recorded the same initial link selection. Agreement was 100% during all concurrent‐chains assessment sessions.

A second observer recorded procedural fidelity during at least 42.86% (*M* = 66.36%) of instructional sessions per participant and 39.13% (*M* = 51%) of probes per participant. The observer recorded fidelity for each target presented during instructional sessions and probe sessions. The percentage of targets presented with procedural fidelity was calculated by dividing the number of targets presented with fidelity by the total number of targets presented and multiplying by 100. During instructional sessions, a trial was implemented with fidelity if the experimenter (a) presented the correct page of the booklet to the participant, (b) allowed at least 5 s for an independent response from the participant, (c) provided the correct condition‐specific consequences following an incorrect response to the target textual stimulus, and (d) delivered praise following a correct response to the target textual stimulus. During probes, the observer scored the trial as being implemented with fidelity if the experimenter (a) presented the probe page to the participant, (b) provided no differential consequences for responses to the target textual stimuli, and (c) provided an echoic prompt contingent on incorrect responses or praise contingent on correct responses to nontarget textual stimuli. Fidelity was 100% for instructional and probe sessions.

An observer recorded procedural fidelity during at least 20% (*M* = 60%) of initial links during the concurrent‐chains assessments per participant. A session was scored as being implemented with fidelity if the experimenter (a) presented the participant with the array of initial links, (b) read the script instructing the participant to select one of the types of books to read, and (c) presented the book that corresponded to the participant's selection. Fidelity was 100% for all initial links.

## RESULTS

**FIGURE 2:**
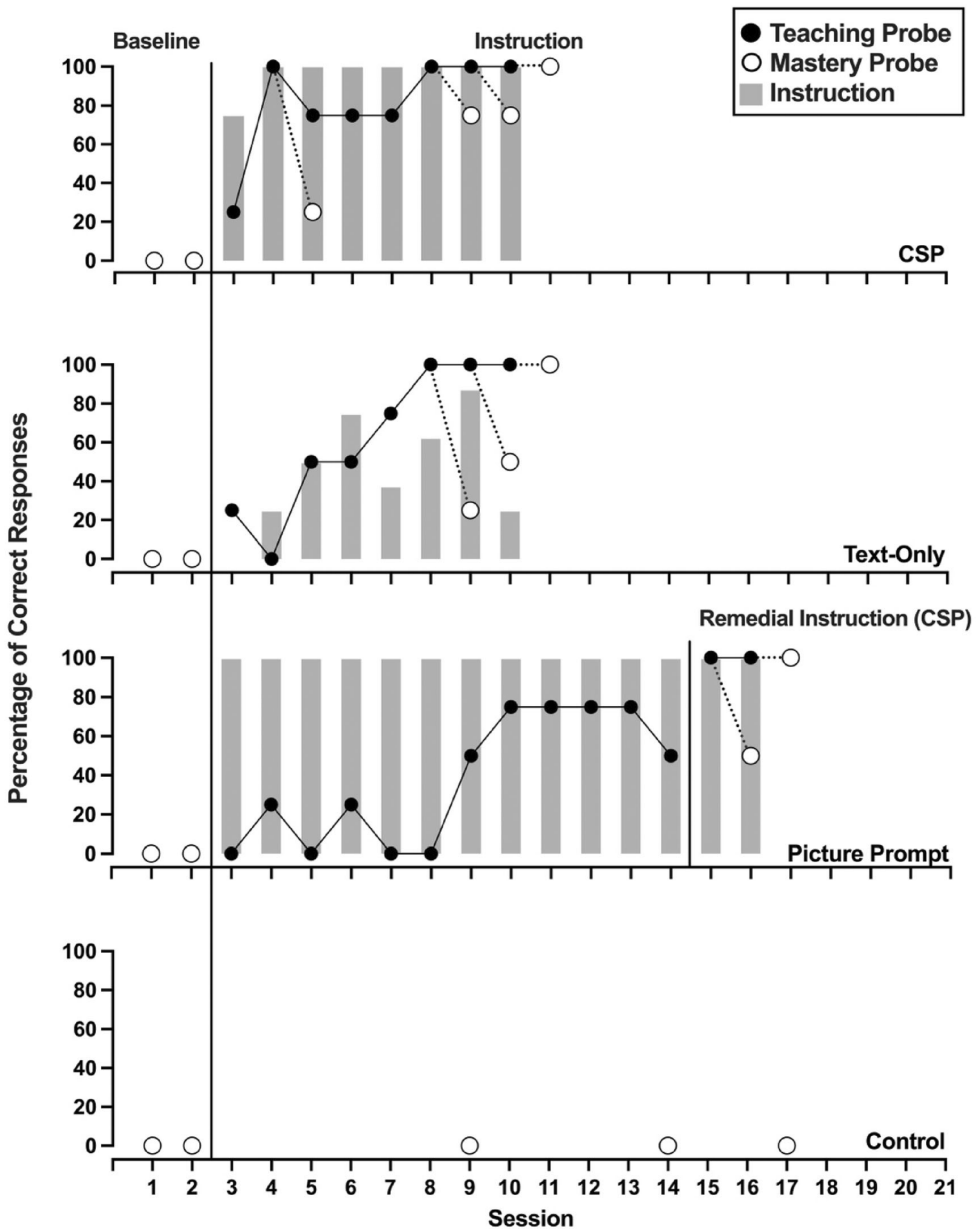
Results of Beau's instructional comparison. The gray bars represent responding while reading the instructional pages of the booklet (i.e., during instruction). One hundred percent correct responding during a teaching probe initiated a mastery probe the following day, which is represented by a dotted line connecting the probes. CSP = compound stimulus prompt.

**FIGURE 3:**
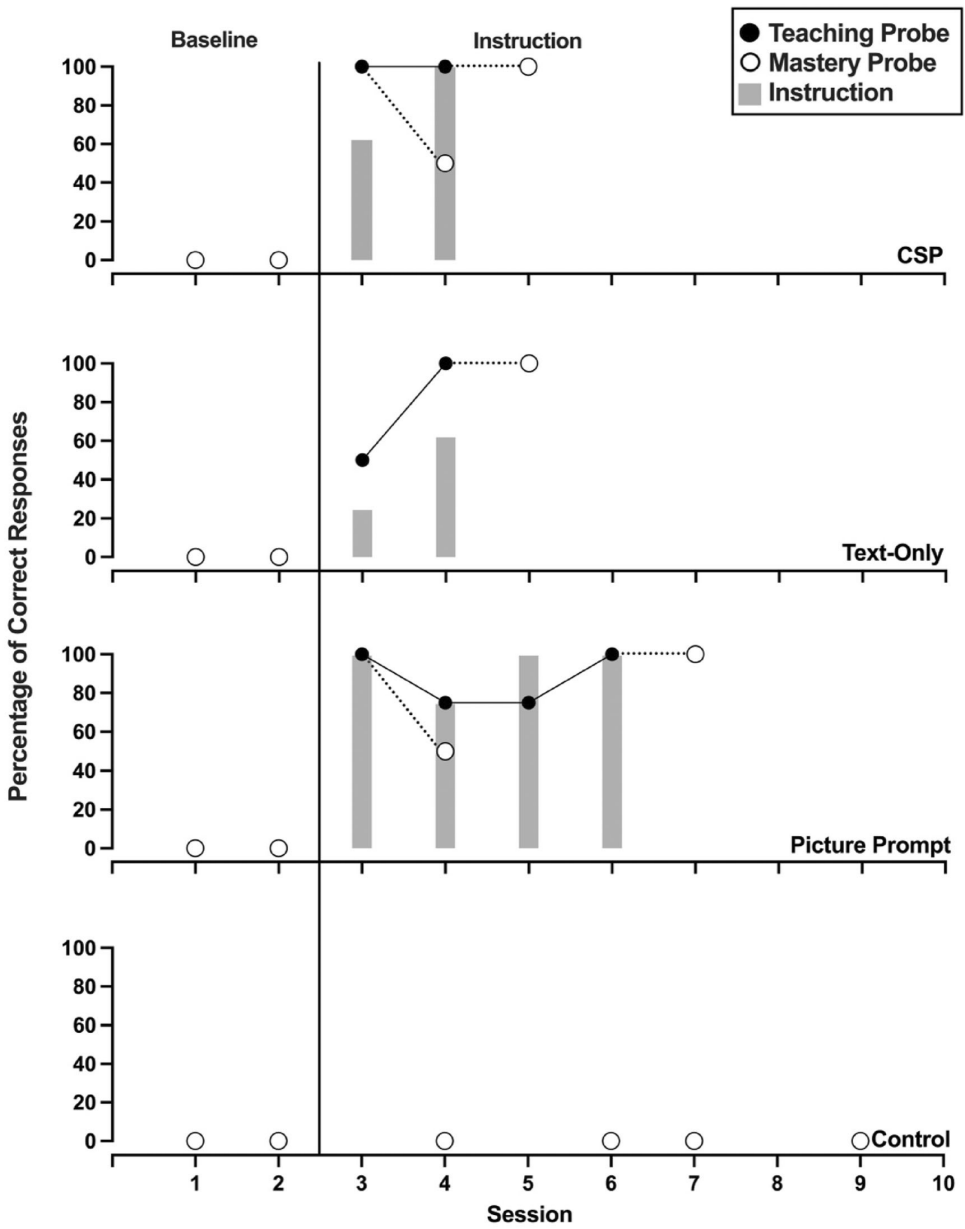
Results of Avina's instructional comparison. The gray bars represent responding while reading the instructional pages of the booklet (i.e., during instruction). One hundred percent correct responding during a teaching probe initiated a mastery probe the following day, which is represented by a dotted line connecting the probes. CSP = compound stimulus prompt.

**FIGURE 4:**
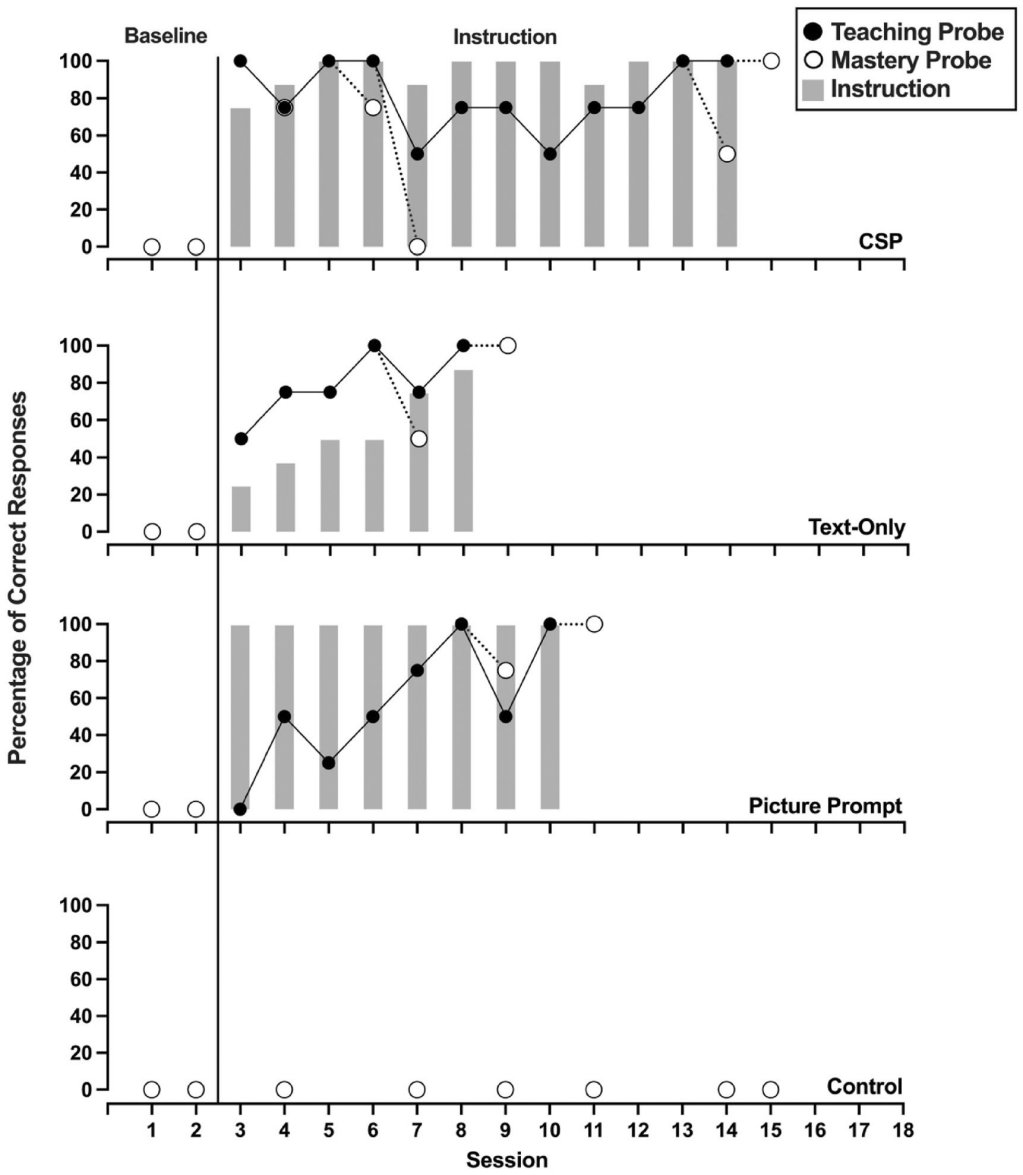
Results of Holly's instructional comparison. The gray bars represent responding while reading the instructional pages of the booklet (i.e., during instruction). One hundred percent correct responding during a teaching probe initiated a mastery probe the following day, which is represented by a dotted line connecting the probes. CSP = compound stimulus prompt.

**FIGURE 5:**
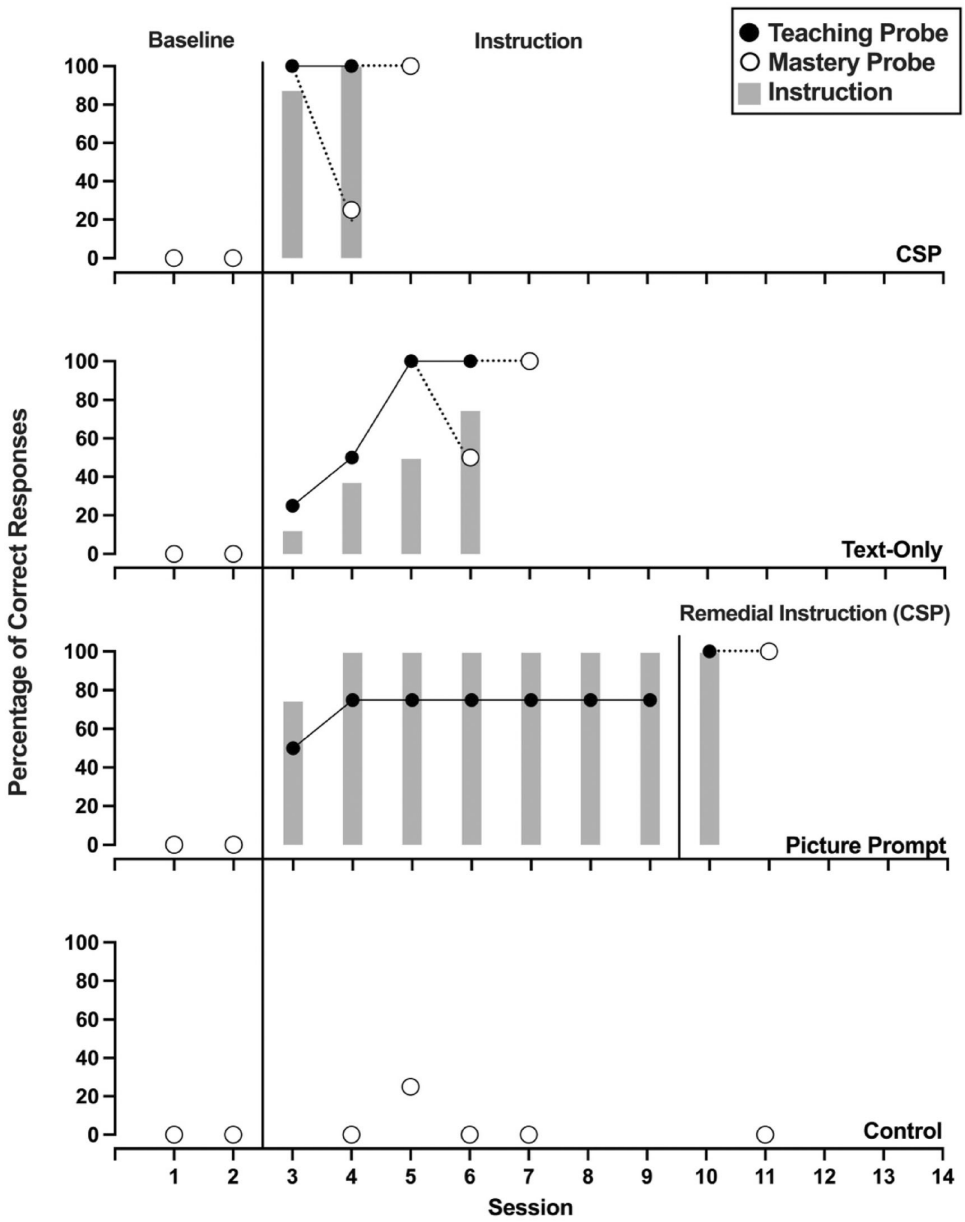
Results of Jalani's instructional comparison. The gray bars represent responding while reading the instructional pages of the booklet (i.e., during instruction). One hundred percent correct responding during a teaching probe initiated a mastery probe the following day, which is represented by a dotted line connecting the probes. CSP = compound stimulus prompt.

**FIGURE 6:**
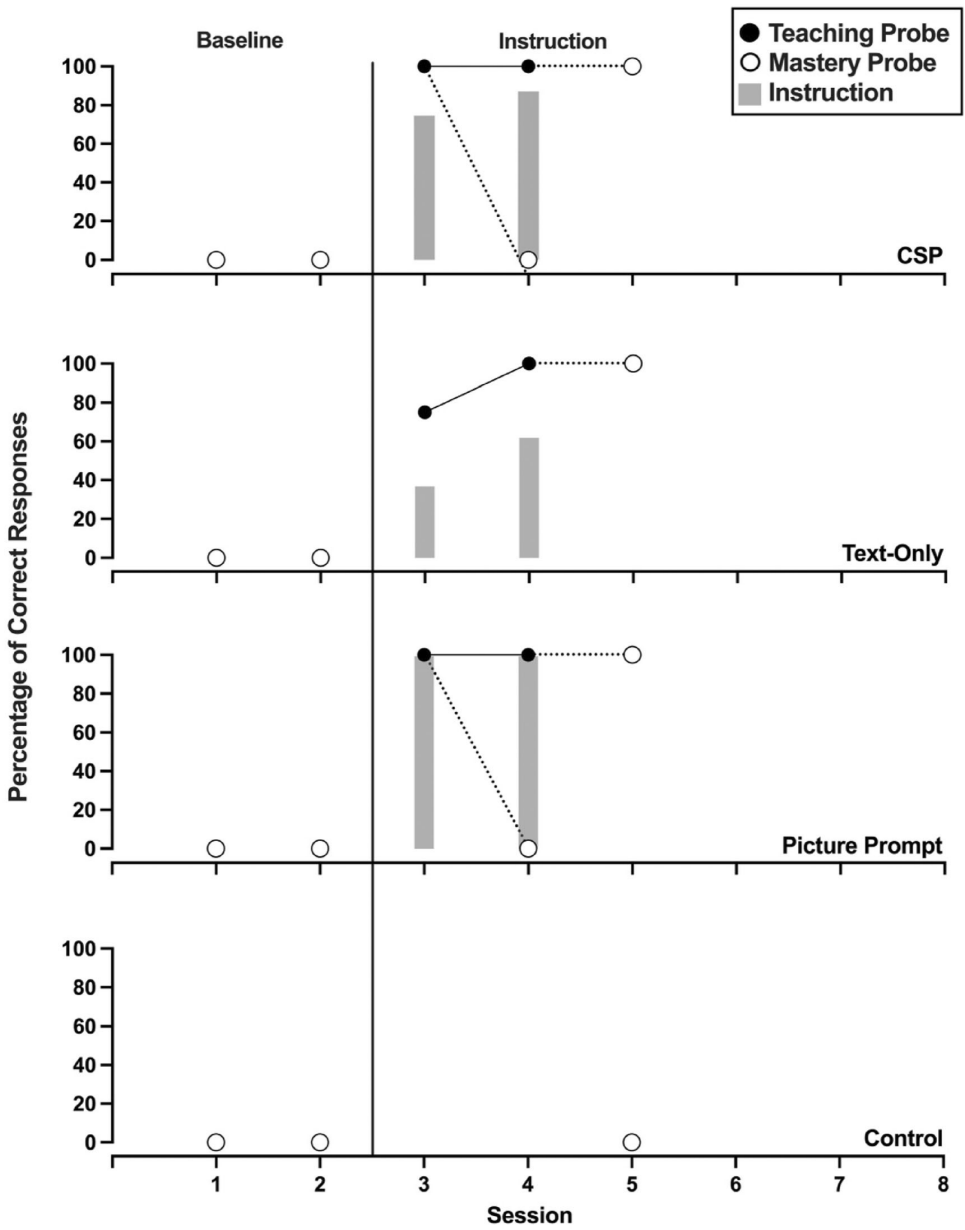
Results of Salem's instructional comparison. The gray bars represent responding while reading the instructional pages of the booklet (i.e., during instruction). One hundred percent correct responding during a teaching probe initiated a mastery probe the following day, which is represented by a dotted line connecting the probes. CSP = compound stimulus prompt.

**FIGURE 7:**
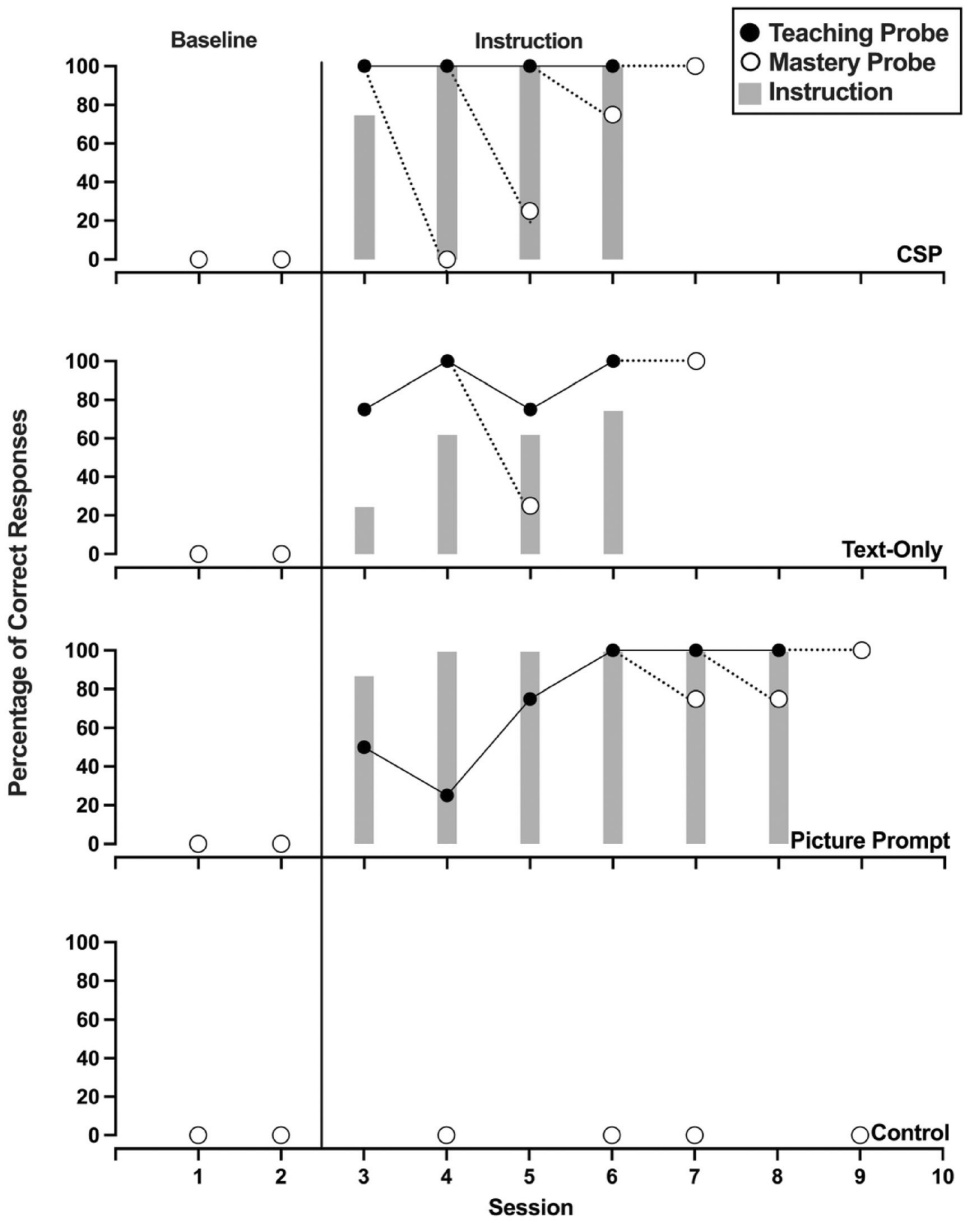
Results of Ivy's instructional comparison. The gray bars represent responding while reading the instructional pages of the booklet (i.e., during instruction). One hundred percent correct responding during a teaching probe initiated a mastery probe the following day, which is represented by a dotted line connecting the probes. CSP = compound stimulus prompt.

Figures 2, 3, 4, 5, 6, 7 show the results of instruction for all participants. The representation of the adapted alternating‐treatments design differs from that of conventional methods such that each condition is presented across rather than within panels. As a reminder, the order of conditions varied each day. No participant responded correctly to targets in any condition during baseline. Only Jalani emitted a single correct response to any target assigned to the control condition.

Figure 2 depicts Beau's comparison. Beau's responding met the mastery criterion in the CSP and text‐only conditions in eight sessions, respectively. Beau initially emitted low levels of correct responding during teaching probes in the picture prompt condition, which increased over time. Nevertheless, his responding did not meet the mastery criterion in the picture prompt condition. We introduced CSP instruction to these targets, and Beau required two sessions before his responding met the mastery criterion. During instruction, Beau responded with high levels of correct responding in the CSP and picture prompt conditions (gray bars). In contrast, he responded with lower levels of correct responding during every instructional session for the text‐only condition. Table 1 shows the total time to mastery for each condition. Beau's responding met the mastery criterion in the fewest minutes in the CSP condition, requiring 10.83 min. In the text‐only condition, Beau required 6.10 min longer to meet the mastery criterion.

**TABLE 1:** Total minutes to mastery, listed as total instructional time for intial and remedial instruction.

Participant	CSP	Picture prompt	Text only
Beau	10.83	13.68^a^	16.93
Avina	2.97	4.48	2.80
Holly	15.48	7.82	10.35
Jalani	3.50	4.58^a^	6.65
Salem	2.92	2.35	2.57
Ivy	5.52	5.48	6.03

^a^ Termination criterion was met prior to mastery.

Figure 3 depicts Avina's comparison. Avina's responding met the mastery criterion in two sessions in both the CSP and text‐only conditions. Her responding met the mastery criterion in the picture prompt condition in four sessions. Avina responded with high levels of correct responding during instruction for the picture prompt and CSP conditions. In the text‐only condition, Avina never emitted greater than 75% correct responses during any instructional session. Table 1 shows Avina's total time to mastery for each condition. Her responding met the mastery criterion in the text‐only condition in 2.80 min and in the CSP condition in 2.97 min. Avina required 4.48 min of instructional time in the picture prompt condition before her responding met the mastery criterion.

Figure 4 shows Holly's comparison. Holly's responding met the mastery criterion in the fewest number of sessions in the text‐only condition, requiring six sessions. Holly's responding met the mastery criterion in eight sessions in the picture prompt condition and 12 sessions in the CSP condition. Holly exhibited 100% correct responding in the picture prompt condition during every instructional session. In the CSP condition, Holly emitted 100% correct responses during all but four instructional sessions. In the text‐only condition, Holly emitted only two correct responses during the first instructional session. Following this session, the percentage of correct responses increased across sessions, although she never emitted 100% correct responses during any instructional session before her responding met the mastery criterion. As shown in Table 1, Holly required the fewest minutes of instructional time in the picture prompt condition (7.82 min). She required 10.35 min in the text‐only condition and 15.48 min in the CSP condition before her responding met the mastery criterion.

Figure 5 depicts Jalani's comparison. Jalani's responding met the mastery criterion following two and four sessions in the CSP and text‐only conditions, respectively. Jalani's responding met the termination criterion in the picture prompt condition following seven sessions. CSP procedures were introduced, which resulted in mastery following a single instructional session. Jalani emitted 87.50% and 100% correct responses during two instructional sessions in the CSP condition. He emitted 100% correct responses in every instructional session in the picture prompt condition, except for one. During the text‐only instructional sessions, Jalani responded correctly to 12.50, 37.50, 50, and 75% of targets. Table 1 shows total minutes to mastery for each condition for Jalani. He required the fewest minutes of instructional time in the CSP condition (3.50 min), followed by the picture prompt (4.58 min) and text‐only conditions (6.65 min).

Figure 6 shows results from Salem's comparison. Salem's responding met the mastery criterion following two sessions for all three conditions. Salem responded correctly to every target during all picture prompt instructional sessions. Salem emitted at least 75% correct responses during both CSP instructional sessions. In contrast, she never emitted greater than 75% correct responses during instruction in the text‐only condition. Table 1 shows Salem's total instructional time. Salem required 2.35, 2.57, and 2.92 min of instruction before her responding met the mastery criterion in the picture prompt, text‐only, and CSP conditions, respectively.

Figure 7 shows Ivy's comparison. Ivy's responding met the mastery criterion for targets in both the CSP and text‐only conditions in four sessions. Her responding met the mastery criterion in the picture prompt condition after six sessions. During instruction in both the CSP and picture prompt conditions, Ivy exhibited 100% correct responding during all but the first sessions. Ivy's correct responding increased across text‐only instructional sessions but never exceeded 75% correct responses during instruction for this condition. Ivy's responding met the mastery criterion in 5.52 and 5.48 min of instruction in the CSP and picture prompt conditions, respectively. She required 6.03 min of instruction in the text‐only condition.

**FIGURE 8:**
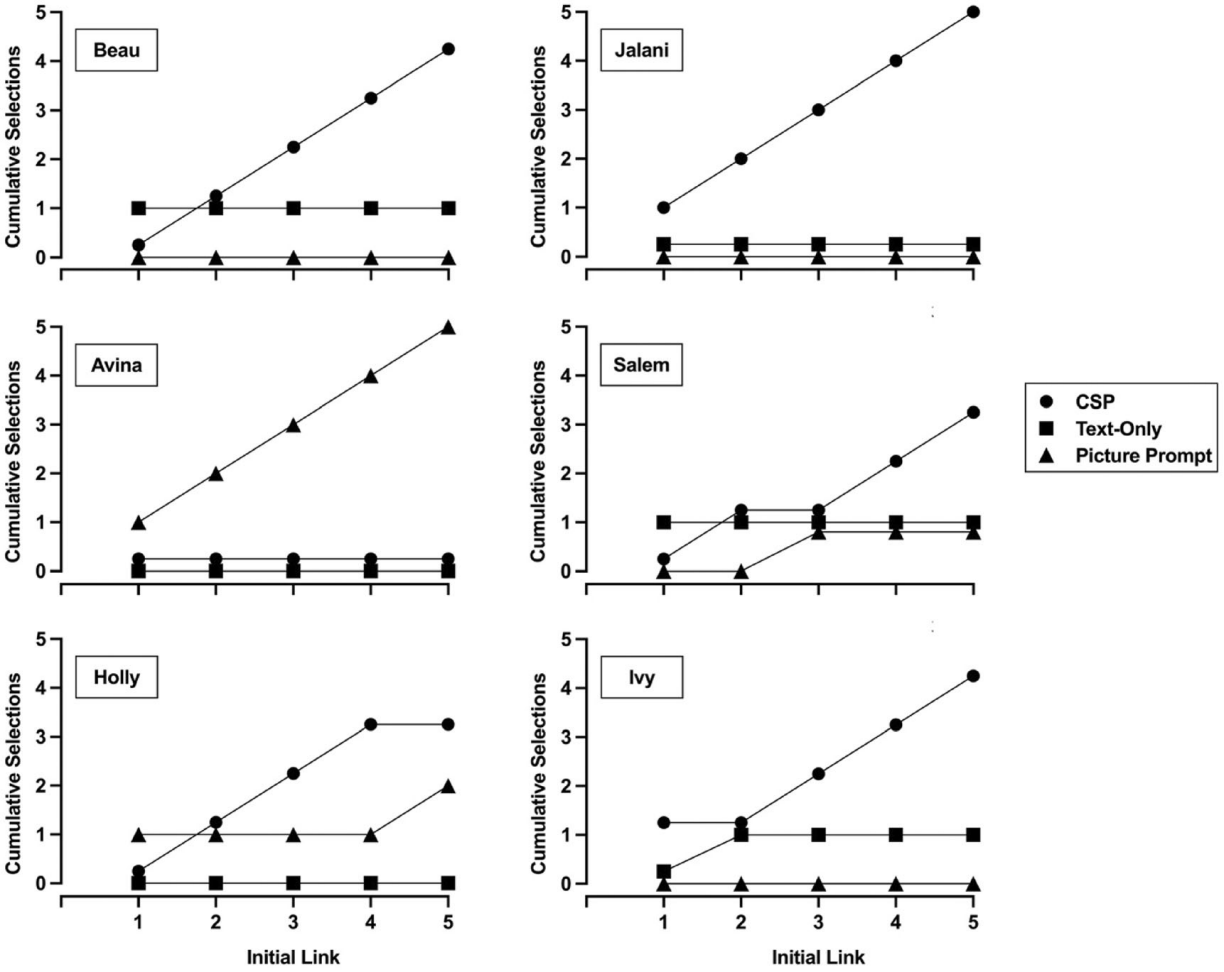
Concurrent‐chains preference assessment outcomes. CSP = compound stimulus prompt.

Figure 8 depicts the findings of each participant's concurrent‐chains preference assessment. Five out of six participants (Beau, Holly, Jalani, Salem, and Ivy) selected the CSP condition during most initial links. Jalani selected the CSP condition during all five initial links. Beau and Ivy selected the CSP condition during four out of five initial links and selected the text‐only condition once. Holly and Salem selected the CSP condition during three out of five initial links. During the remaining two initial links, Holly selected the picture prompt condition twice and Salem selected the picture prompt condition and text‐only condition once each. Avina selected the picture prompt condition during all five initial links.

## DISCUSSION

We evaluated the effect of three conditions (i.e., CSP, text‐only, and picture prompt) on the development of control by textual stimuli during picture‐book reading. Our results support previous research suggesting that pictures inhibit control by textual stimuli when the two elements are presented simultaneously (Didden et al., 2000; Richardson et al., 2017; Samuels, 1967; Singh & Solman, 1990; see review by Kennedy & Cariveau, 2023). Specifically, four of the six participants required the greatest number of sessions to meet the mastery criterion in the picture prompt condition. Furthermore, the current study is the first to find that a condition that included picture stimuli and a text‐only condition were similarly effective in producing textual control. The CSP condition produced mastery‐level responding for all participants, with five out of six requiring the same or fewer sessions to meet the mastery criterion in the CSP condition relative to the text‐only condition.

Previous research examining methods to remediate the picture‐text problem has generally found that text‐only instruction produced responding controlled by textual stimuli more rapidly than instruction that included pictures (Didden et al., 2000; Richardson et al., 2017; Singh & Solman, 1990; Wu & Solman, 1993). In the current study, we hypothesized that the CSP condition required that the learner differentially respond to the textual element of the compound stimulus. Requiring a response to the underselected element (i.e., the textual stimulus; Broomfield et al., 2008) effectively strengthened control by all relevant features of the presented picture–text compound. The current findings align with those of recent research demonstrating the effectiveness of the CSP arrangement (Lewis et al., 2024) and suggests that this arrangement may also effectively remediate the picture‐text problem in book reading. Beyond efficiency, additional findings of the current study further support the superiority of the CSP condition, as this condition resulted in higher levels of correct responding during instruction and was preferred by five participants during the concurrent‐chains assessment.

The CSP and picture prompt conditions resulted in higher levels of correct responding during instruction relative to the text‐only condition. Indeed, no participants in the current study emitted 100% correct responses to targets during any instructional session in the text‐only condition. In contrast, each participant emitted at least 75% correct responses during all CSP teaching sessions except for Avina's first session. During the concurrent‐chains assessment, each participant preferred the CSP or picture prompt conditions to the text‐only condition. In fact, the text‐only condition was selected during only three out of 30 total initial links during this assessment. The coherence between participants' preference and low levels of incorrect responding during instruction for conditions that included pictures further bolster support for the CSP condition in the current study. These findings suggest that the CSP arrangement may be an optimal method for remediating the picture‐text problem in book reading for children who exhibit reading deficits.

Future research may address the limitations of this study. First, the current study did not include within‐participant replication of intervention effects, which may be relevant when considering idiosyncratic findings across participants. Specifically, the findings from Holly's comparison differed such that the CSP condition required the greatest number of sessions to produce responding at the mastery criterion. Conducting additional within‐participant comparisons might allow for greater confidence in the consistency of the observed effects or detection of other sources of variability (Cariveau & Fetzner, 2022; Sidman, 1960). Additionally, the findings of Salem's evaluation revealed equivocal outcomes across conditions. Salem's findings suggest that pictures may not always impede the development of textual control. Future research should consider how to best characterize participants' learning histories that may evince reduced susceptibility to conditions that commonly produce restricted stimulus control.

A second limitation of the current study relates to the ecological validity of the CSP book arrangements. The ecological validity of a study, or the degree to which the materials and methods used align with those of the natural environment (e.g., home environment or school), should be considered (Fahmie et al., 2023). Although the CSP condition effectively remediated the picture‐text problem and produced nearly errorless responding during instruction, the current arrangement of the picture–text compound arrays differs from how pictures appear in typical reading materials. Additionally, although five of the six participants preferred the CSP condition, it is unclear whether this arrangement would be similarly preferred by caregivers or teachers. Future research should assess other stakeholders' preference for these arrangements and should also assess the efficacy of similar compound stimulus prompts in reading conditions that mimic those of other popular picture books. For example, arranging larger picture–text compound arrays, similar to those in *I Spy* books, or providing participants with a picture‐text dictionary that can be used during reading instruction may be as effective and socially valid as the current CSP condition.

This study extended previous research on the picture‐text problem by (a) assessing the effects of picture‐text arrangements on the development of textual control during picture‐book reading, (b) evaluating a novel prompting method to remediate or prevent the picture‐text problem in picture‐book reading, and (c) assessing participants' preference for the conditions (i.e., book types). The findings of this study suggest several possible lines of research for those interested in remediating restricted stimulus control with compound stimuli. Although previous research on the picture‐text problem has suggested that removing pictures from instruction may be required, the current findings suggest that the effectiveness of the CSP arrangement was similar to or greater than that with text‐only instruction, was more preferred, and resulted in higher levels of correct responding during instruction. The current findings may embolden behavior analysts to consider how stimulus control technologies can be used to improve learning outcomes while prioritizing the use of preferred instructional arrangements.

## AUTHOR CONTRIBUTIONS

T. K. L. conceived the project, developed the methodology, collected data, and contributed to the writing of the original draft and revisions. T.C. contributed to the development of the methodology, collected data, provided supervision, and reviewed and edited the manuscript.

## CONFLICT OF INTEREST STATEMENT

The authors have no conflicts of interest to disclose.

## ETHICS APPROVAL

This research was approved by the Institutional Review Board at the University of North Carolina Wilmington. Informed consent was obtained by legal guardians before participation began and vocal assent was obtained by each participant every day before instruction.

## Data Availability

The data that support the findings of this study are available from the corresponding author upon reasonable request.
